# Risk Factors, Preventive Practices, and Health Care Among Breast Cancer Survivors, United States, 2010

**DOI:** 10.5888/pcd13.150377

**Published:** 2016-01-21

**Authors:** Sherri G. Homan, Noaman Kayani, Shumei Yun

**Affiliations:** Author Affiliations: Noaman Kayani, Missouri Department of Health and Senior Services, Jefferson City, Missouri; Shumei Yun, Missouri Department of Health and Senior Services, Jefferson City, Missouri, and University of Missouri, Columbia, Missouri. Sherri Homan is also affiliated with the University of Missouri, Sinclair School of Nursing, Columbia, Missouri.

## Abstract

**Introduction:**

We compared behavioral risk factors and preventive measures among female breast cancer survivors, female survivors of other types of cancers, and women without a history of cancer. Survivorship health care indicators for the 2 groups of cancer survivors were compared.

**Methods:**

Using data from the 2010 Behavioral Risk Factor Surveillance System, we calculated the proportion of women with risk factors and their engagement in preventive practices, stratified by cancer status (cancer survivors or women with no history of cancer), and compared the proportions after adjusting for sociodemographic characteristics.

**Results:**

A significantly higher proportion of breast cancer survivors had mammography in the previous year (79.5%; 95% confidence interval [CI], 76.0%–83.0%) than did other cancer survivors (68.1%; 95% CI, 65.6%–70.7%) or women with no history of cancer (66.4%; 95% CI, 65.5%–67.3%). Breast cancer survivors were also more likely to have had a Papanicolaou (Pap) test within the previous 3 years than women with no history of cancer (89.4%; 95% CI, 85.9%–93.0 vs 85.1%; 95% CI, 84.4%–85.8%) and a colonoscopy within the previous 10 years (75.4%; 95% CI, 71.7%–79.0%) than women with no history of cancer (60.0%; 95% CI, 59.0%–61.0%). Current smoking was significantly lower among survivors of breast cancer (10.3%; 95% CI, 7.4%–13.2%) than other cancer survivors (20.8%; 95% CI, 18.4%–23.3%) and women with no history of cancer (18.3%; 95% CI, 17.5%–19.1%). After adjusting for sociodemographic characteristics, we found that breast cancer survivors were significantly more likely to have had mammography, a Pap test, and colonoscopy, and less likely to be current smokers.

**Conclusion:**

Breast cancer survivors are more likely to engage in cancer screening and less likely to be current smokers than female survivors of other types of cancer or women with no history of cancer.

## Introduction

In 2012, there were approximately 13.8 million cancer survivors in the United States, of which 21.6% were female breast cancer survivors (1). Although the incidence of breast cancer among US women has been relatively stable during the past decade, deaths from breast cancer have declined on average 1.9% each year from 2002 through 2011. Increased rates of breast cancer survivorship bring with them potential health challenges (2), including recurrence, related to a previous breast cancer (3–5) and development of a second primary cancer, a new cancer with a different histology than a previous tumor (6–8). In a large surveillance study, researchers found that, on average, 12.3% of breast cancer survivors had a second primary cancer, and this risk varied by age and cancer type with a mean time until the second cancer of 6.2 years (8 years for patients with breast cancer diagnosed prior to age 50 and 5.7 years for patients with breast cancer diagnosed at or older than age 50) (6). Excess risks for 13 second primary cancers were found among breast cancer survivors. The leading sites of second cancers on the basis of total cases (N = 44,249) were breast (n = 22,927; 51.8%), colorectal (n = 4,321; 9.8%), and lung (n = 3,507; 7.9%) cancers (6).

Breast cancer recurrence and development of second primary cancers are affected by genetic (4,7,9) or hormonal factors (3), tumor characteristics (10), treatments (3,4,7,8), and certain modifiable risk factors (11,12) such as smoking, obesity, and alcohol consumption. Engaging in physical activity may reduce the risk of cancer recurrence and all-cause mortality (13,14). Providing patients with screening recommendations and guidance about health promotion activities are part of high-quality survivorship care (15). However, only limited information exists on the prevalence of behavioral risk factors among breast cancer survivors. This information is crucial to planning for the best survivorship care for optimal health results.

The purpose of this study was to compare the prevalence estimates of behavioral risk factors and preventive practices, including cancer screening, for female breast cancer survivors, female survivors of other types of cancer, and women with no history of cancer. We also examined survivorship health care to inform future public health and health care planning and care management strategies.

## Methods

The Behavioral Risk Factor Surveillance System (BRFSS) is a standardized, random-digit–dialed telephone survey of noninstitutionalized US adults, implemented by health agencies in all 50 states, the District of Columbia, and 3 US territories. The primary purpose of the BRFSS is to provide state-specific estimates of the prevalence of self-reported behavioral risk factors associated with the leading causes of death and disability in the United States. The BRFSS questionnaire includes a set of core questions, optional modules, and state-specific questions. Cancer screening questions are included in the core questionnaire, and the 2010 BRFSS also offered a cancer survivorship optional module. The data were weighted using post stratification methodology to adjust for the unequal probability of selection, differential nonresponse, and possible deficiencies in the sampling frame (16,17).

The study population consisted of 39,235 women aged 18 years or older who participated in the 2010 BRFSS in 1 of the 9 states and 1 territory that implemented the cancer survivorship optional module. The women were stratified into 3 groups: 1) women with a history of breast cancer (n = 1,310), 2) female survivors of a type of cancer other than breast cancer (n = 3,556), and 3) women with no history of cancer (n = 34,369). The study was limited to women with a single type of cancer.

Race and ethnicity were summarized in 4 categories: non-Hispanic white, non-Hispanic African American, non-Hispanic other race, and Hispanic. Education attainment was grouped into 3 categories: less than a high school education, high school graduate, or greater than a high school education. Age in years was grouped into 3 categories: less than 50 years, 50 to 64 years, and 65 years or older. Annual household income was grouped into 5 categories: less than $15,000, $15,000 to less than $25,000, $25,000 to less than $35,000, $35,000 to less than $50,000, and $50,000 or greater. Disability was based on a yes response to the question “Are you limited in any activities because of physical, mental, or emotional problems?” Health care coverage was assessed as a yes response to the question “Do you have any kind of health care coverage, including private health insurance, prepaid plans such as HMOs [health maintenance organizations], or government plans such as Medicare?”

Current smokers were defined as having smoked 100 cigarettes during their lifetime and who currently smoke some days or every day. Weight status was defined by body mass index (BMI), calculated as weight in kilograms divided by the square of height in meters (kg/m^2^): underweight or normal (BMI <25 kg/m^2^), overweight (BMI 25–29.9 kg/m^2^), or obese (BMI ≥30 kg/m^2^). No leisure-time physical activity was derived from a no response to the question “During the past 30 days, other than your regular job, did you participate in any physical activities or exercise such as running, calisthenics, golf, gardening, or walking for exercise?” Heavy drinking was defined as having more than 1 alcoholic drink per day. The BRFSS cancer screening tests assessed were mammography, clinical breast examination (CBE), Papanicolaou (Pap) test, blood stool test, sigmoidoscopy or colonoscopy, and length of time since most recent examinations. Comorbid conditions considered for potential confounders were diabetes, angina, and myocardial infarction.

As part of the BRFSS cancer survivorship optional module, respondents were asked whether they had ever been told by a doctor, nurse, or other health professional that they had cancer, and if yes, the number of different cancers, and age at diagnosis. Women with a diagnosis of a single type of cancer were selected and grouped into 4 categories by age at diagnosis: less than 40 years, 40 to 49 years, 50 to 79 years, and 80 years or older. Time since diagnosis in years was calculated and also grouped into 4 categories: less than 5 years, 5 to 9 years, 10 to 19 years, and 20 or more years. All cancer survivors were asked if they were currently receiving treatment. Women not currently in treatment were asked what type of doctor provided most of their health care, whether they had ever been given a written summary of cancer treatments received or instructions for routine cancer check-ups, whether they ever participated in a clinical trial as part of their treatment, whether health insurance paid for all or part of treatment costs or if they were ever denied insurance because of cancer, and current physical pain related to the cancer or treatment and pain control status.

### Statistical analysis

We calculated the proportion of women with risk factors for cancer and their engagement in preventive practices stratified by cancer status — breast cancer survivors, female survivors of other cancers, and women without a history of cancer. Using multivariate logistic regression, we compared the proportions of the 3 groups by adjusting for age, race, education, and household income of participants. We also controlled for selected comorbid conditions and general disability to assess the impact on the model. We further compared indicators of survivorship health status for the 2 survivor groups and assessed the impact of time since diagnosis on leisure-time physical inactivity and current physical pain. Statistical significance was determined by 95% confidence intervals (CIs) for the prevalence or adjusted odds ratios (AORs). We used the χ^2^ test to compare the distributions of age at diagnosis and time since diagnosis between the breast cancer survivors and other cancer survivors. The Missouri Department of Health and Senior Services Institutional Review Board found this study to be exempt from review. Data were analyzed by using SAS 9.4 (SAS Institute, Inc).

## Results

Breast cancer survivors tended to be older (55.8% ≥ age 65) than other cancer survivors (39.4%) and women with no history of cancer (17.2%) (Table 1). Most women in all 3 groups reported completing high school or had a higher than high school education. A high proportion of women in each group had a household income of $50,000 or more (39.7% for breast cancer survivors; 38.1% survivors of other cancer; 49.7% for women with no history of cancer), and the proportion was more than 10 percentage points higher for women with no history of cancer. Most of the women had health insurance coverage. Among cancer survivors, the most frequently reported age at diagnosis was between age 50 and 79 (59.2% breast; 40.6% other cancer survivors); however, the female survivors of other types of cancer tended to be younger when the cancer was diagnosed; 40.2% were given a diagnosis before age 40. A small proportion of women survivors of other types of cancer (1.2%) and even fewer breast cancer survivors (0.2%) reported receiving their diagnosis before age 18. The time since diagnosis differed significantly between the 2 cancer survivorship groups. A larger proportion of female survivors of other cancer types (21.6%) had 20 years or more since diagnosis compared with breast cancer survivors (14.8%) (*P* < .001).

**Table 1:** Characteristics of Female Breast Cancer Survivors, Female Survivors of Other Cancer Types, and Women With No History of Cancer, Behavioral Risk Factor Surveillance System, 9 US States and 1 US Territory^a^, 2010, (N = 39,235)

Characteristic	Breast Cancer Survivors^b^	Female Survivors of Other Cancer Types^b^^,^ c	Women With No History of Cancer	
	N^d^(%)^e^	N^d^(%)^e^	N^d^(%)^e^	
**Overall**	1,310 (100)	3,556 (100)	34,369 (100)	
**Race**				
Non-Hispanic white	1,122 (87.8)	3,091 (89.3)	26,726 (81.9)	
Non-Hispanic African American	67 (5.5)	145 (4.0)	2,624 (7.3)	
Non-Hispanic other	56 (3.4)	138 (3.9)	2,033 (4.7)	
Hispanic	56 (3.3)	141 (2.7)	2,594 (6.0)	
**Age group, y **				
<50	94 (12.5)	605 (30.7)	13,114 (60.5)	
50–64	388 (31.6)	1,096 (29.9)	10,575 (22.4)	
≥65	810 (55.8)	1,826 (39.4)	10,267 (17.2)	
**Education**				
<High school	95 (5.6)	358 (9.3)	3,034 (7.0)	
High school	415 (31.4)	1,170 (32.6)	10,453 (29.1)	
>High school	798 (63.0)	2,015 (58.1)	20,800 (63.8)	
**Household income, $**				
<15,000	133 (9.7)	438 (12.4)	3,549 (8.4)	
15,000–24,999	226 (17.5)	697 (21.4)	5,512 (15.9)	
25,000–34,999	166 (16.3)	392 (13.8)	3,663 (12.0)	
35,000–49,999	155 (16.7)	420 (14.2)	4,163 (13.9)	
≥50,000	361 (39.7)	926 (38.1)	11,950 (49.7)	
**Age at cancer diagnosis, y^f^**				
<40	119 (12.7)	999 (40.2)	—	—
40–49	232 (21.5)	450 (13.3)	—	—
50–79	862 (59.2)	1,782 (40.6)	—	—
≥80	94 (6.6)	282 (5.8)	—	—
**Time since cancer diagnosis, y^f^**				
<5	418 (31.3)	1,222 (34.7)	—	—
5–9	299 (27.2)	655 (18.9)	—	—
10–19	375 (26.7)	827 (24.8)	—	—
≥20	218 (14.8)	851 (21.6)	—	—
**Disability**	450 (34.6)	1,320 (37.3)	8,615 (25.2)	
**No health insurance (aged 18–64 y)**	30 (4.5)	159 (11.5)	2,812	(12.9)

Abbreviation: —, not applicable.

a The 9 states are Alaska, Connecticut, Indiana, Massachusetts, Missouri, New Mexico, Ohio, South Dakota, and Wisconsin, and the US territory is the Virgin Islands.

b Women with 1 cancer only, excludes women with multiple types of cancer.

c Excludes breast cancer survivors.

d Denominator varies with missing and unknown excluded.

e May not sum to 100 because of rounding.

f Significant difference in the distribution of age at diagnosis and time since cancer diagnosis, χ^2^ testing (*P* < .001).

### Survivorship health care

A significantly larger proportion of breast cancer survivors (16.5%; 95% CI, 13.3%–19.6%) than survivors of other cancers (6.9%; 95% CI, 5.6%–8.3%) was currently receiving treatment. Of the survivors not currently receiving treatment, primary care doctors were reported as providing most health care for breast cancer survivors (73.7%; 95% CI, 69.4%–78.0%) and other cancer survivors (72.8%; 95% CI, 70.4%–75.3%) (Table 2). Although approximately one-third of the cancer survivors received a written summary of cancer treatments, a significantly greater proportion of breast cancer survivors (39.3%; 95% CI, 34.7%–43.8%) than other cancer survivors (27.8%; 95% CI, 25.2%–30.4%) received such a summary. A significantly greater proportion of breast cancer survivors (80.0%; 95% CI, 76.2%–83.8%) than other cancer survivors (67.9%; 95% CI, 65.3%–70.6%) received instructions on routine cancer check-ups; fewer in either survivor group received these instructions in a written or printed format (62.3%; 95% CI, 56.8%–67.9% breast cancer vs 63.7%; 95% CI, 60.0%–67.4% other cancer).

**Table 2:** Comparison of Health Care Indicators for Breast Cancer Survivors and Female Survivors of Other Cancer Types, Behavioral Risk Factor Surveillance System, 9 US States^a^ and 1 US Territory, 2010 (N = 4,866)

Health Care Indicator	Breast Cancer Survivors[Table-fn T2FN2]	Female Survivors of Other Cancer Types^b^^,^ c
	Percentage (95% CI) [n]^d^	Percentage (95% CI) [n]^d^
**Overall n (%)**	1,310 (100)	3,556 (100)
**Currently receiving treatment** **(Surgery, radiation therapy, chemotherapy or oral chemotherapy)**	16.5 (13.3–19.6) [208]	6.9 (5.6–8.3) [264]
**Doctor providing majority of health care^e^^,^ f**		
Oncologist	10.1 (7.1–13.1) [105]	8.5 (7.1–10.1) [256]
Surgeon	5.6 (3.4–7.8) [68]	4.8 (3.6–6.0) [159]
Primary care	73.7 (69.4–78.0) [802]	72.8 (70.4–75.3) [2,314]
Other	10.6 (7.7–13.6) [94]	13.8 (11.9–15.6) [457]
**Patient received written summary of cancer treatments^f^^,^ g**	39.3 (34.7–43.8) [401]	27.8 (25.2–30.4) [818]
**Patient received instructions on routine cancer check-ups of where to return or whom to see^f^^,^ g**	80.0 (76.2–83.8) [837]	67.9 (65.3–70.6) [2,073]
**Patient received written or printed instructions on routine cancer check-ups^f^^,^ g**	62.3 (56.8–67.9) [485]	63.7 (60.0–67.4) [1,194]
**Health insurance paid for all or part of cancer treatment^f^**	97.4 (96.0–98.8) [1,041]	92.9 (91.2–94.6) [2,965]
**Ever denied health insurance because of cancer diagnosis^f^**	8.1 (5.9–10.5) [102]	7.6 (6.0–9.1) [227]
**Participated in clinical trial^f^**	8.1 (5.8–10.3) [98]	4.2 (2.9–5.4) [110]
**Current physical pain due to cancer or cancer treatment^f^**	15.1 (11.4–18.8) [128]	7.5 (6.0–9.0) [201]
**Current physical pain by time since diagnosis, y f ^,^ h**		
<5	18.8 (11.3–26.3) [43]	11.6 (8.4–14.9) [93]
5–9	21.2 (13.0–29.5) [41]	6.6 (3.8–9.4) [48]
10–19	10.4 (5.3–15.5) [31]	7.3 (4.1–10.6) [38]
≥20	7.1 (1.3–13.0) [13]	2.6 (1.0–4.1) [22]
**Pain not currently under control^f^^,^ i**	8.8 (1.9–15.6) [14]	20.3 (12.0–28.5) [37]

a The 9 states are Alaska, Connecticut, Indiana, Massachusetts, Missouri, New Mexico, Ohio, South Dakota, and Wisconsin, and the US territory is the Virgin Islands.

b Women with 1 cancer only, excludes women with multiple types of cancer.

c Excludes breast cancer survivors.

d Missing and unknown excluded.

e May not sum to 100 because of rounding.

f Excludes survivors undergoing treatment.

g Ever received from doctor, nurse, or other health professional.

h Significant decline breast cancer survivors (*P* < .001) and other survivors (*P* < .001).

i Among women with current physical pain due to cancer or cancer treatment.

Health insurance paid for all or part of the cancer treatments of the majority (>90%) of cancer survivors in both groups. A small proportion of breast cancer survivors (8.1%; 95% CI, 5.9%–10.5%) and other cancer survivors (7.6%; 95% CI, 6.0%–9.1%) had been denied health insurance because of a cancer diagnosis. A small but significantly greater proportion of breast cancer survivors had participated in a clinical trial (8.1%; 95% CI, 5.8%–10.3%) compared with other cancer survivors (4.2%; 95% CI, 2.9%–5.4%). A significantly higher proportion of breast cancer survivors (15.1%; 95% CI, 11.4%–18.8%) than other cancer survivors (7.5%; 95% CI, 6.0%–9.0%) reported current physical pain related to the cancer or its treatment; however, as length of time since diagnosis increased, the proportions of survivors reporting pain significantly declined for breast cancer survivors (*P* < .001) and female survivors of other cancer types (*P* < .001). A smaller proportion of breast cancer survivors (8.8%; 95% CI, 1.9%–15.6%) than other cancer survivors (20.3%; 95% CI, 12.0%–28.5%) reported their pain as not under control.

Current smoking was significantly lower among breast cancer survivors (10.3%; 95% CI, 7.4%–13.2%) than among other female cancer survivors (20.8%; 95% CI, 18.4%–23.3%) and women with no history of cancer (18.3%; 95% CI, 17.5%–19.1%) (Table 3). After adjusting for sociodemographic characteristics, we found that other female cancer survivors were significantly more likely than breast cancer survivors to be current smokers (AOR = 1.56; 95% CI, 1.04–2.32). The prevalence of overweight and obesity was not significantly different among the 3 groups. However, in the risk categories, a significantly higher proportion of breast cancer survivors (34.1%; 95% CI, 30.1%–38.1%) did not engage in leisure-time physical activity compared with women with no history of cancer (24.7%; 95% CI, 23.9%–25.5%), and this difference remained significant after we adjusted for sociodemographic characteristics (AOR = 0.73; 95% CI, 0.59–0.90) (Table 3) and persisted after we adjusted for disability (AOR = 0.77; 95% CI, 0.63–0.95). Similar to breast cancer survivors, one-third (32.7%); AOR = 0.95; 95% CI, 0.72–1.17) of female survivors of other types of cancer also reported no leisure time physical activity. Adjusting for time since diagnoses had little effect on the AOR for physical activity (AOR = 0.91; 95% CI, 0.71–1.17) beyond the socioeconomic variables. Heavy drinking was low among all 3 groups: 3.6% (95% CI, 2.3%–5.0%) for breast cancer survivors, 4.5% (95% CI, 3.4%–5.6%) for other cancer survivors, and 4.4% (95% CI, 4.1%–4.8%) for women with no history of cancer. Differences between groups were not significant.

**Table 3:** Prevalence of Selected Risk Factors and Cancer Screenings Among 3 Groups of Women: Breast Cancer Survivors^a^, Female Survivors of Other Cancer Types^a^^,^ b, and Women With No History of Cancer, Behavioral Risk Factor Surveillance System, 9 US States and 1 Territory^c^, 2010 (N = 39,235)

Risk Factors and Cancer Screening	% (95% CI) [n^d^]	Odds Ratio (95% CI)	Adjusted OR^e^ (95% CI)
**Risk factors**			
**Current smoker** f			
Breast cancer survivors	10.3 (7.4–13.2) [1,307]	1 [Reference]	
Female survivors of other cancers	20.8 (18.4–23.3) [3,542]	2.28 (1.62–3.21)	1.56 (1.04–2.32)
Women with no history of cancer	18.3 (17.5–19.1) [34,201]	1.95 (1.43–2.66)	1.18 (0.81–1.71)
**Overweight (BMI 25.0–29.9)**			
Breast cancer survivors	33.7 (29.6–37.8) [1,229]	1 [Reference]	
Female survivors of other cancers	29.6 (27.1–32.0) [3,366]	0.83 (0.66–1.03)	0.95 (0.74–1.21)
Women with no history of cancer	29.1 (28.2–29.9) [31,989]	0.81 (0.67–0.97)	1.03 (0.83–1.28)
**Obese (BMI ≥30.0)**			
Breast cancer survivors	26.6 (22.5–30.8) [1,229]	1 [Reference]	
Female survivors of other cancers	29.1 (26.6–31.7) [3,366]	1.13 (0.89–1.45)	1.04 (0.79–1.38)
Women with no history of cancer	26.7 (25.8–27.5) [31,989]	1.00 (0.81–1.24)	0.93 (0.73–1.20)
**No leisure-time physical activity in previous month**			
Breast cancer survivors	34.1 (30.1–38.1) [1,308]	1 [Reference]	
Female survivors of other cancers	32.7 (30.2–35.3) [3,549]	0.94 (0.76–1.16)	0.95 (0.72–1.17)
Women with no history of cancer	24.7 (23.9–25.5) [34,332]	0.63 (0.53–0.76)	0.73 (0.59–0.90)
**Heavy drinking** g			
Breast cancer survivors	3.6 (2.3–5.0) [1,275]	1 [Reference]	
Female survivors of other cancers	4.5 (3.4–5.6) [3,493]	1.25 (0.79–1.99)	1.24 (0.74–2.08)
Women with no history of cancer	4.4 (4.1–4.8) [33,813]	1.24 (0.83–1.84)	1.23 (0.79–1.92)
**Cancer screening**			
**Breast cancer screening^h^**			
**Ever had mammography**			
Breast cancer survivors	99.8 (99.5–100.0) [1,281]	1 [Reference]	
Female survivors of other cancers	95.7 (94.5–96.9) [3,331]	0.05 (0.02–0.19)	0.05.(0.01–0.26)
Women with no history of cancer	91.3 (90.8–91.9) [27,495]	0.03 (0.01–0.09)	0.03 (0.01–0.14)
**Last mammography** < **1 year**			
Breast cancer survivors	79.5 (76.0–83.0) [1,266]	1 [Reference]	
Female survivors of other cancers	68.1 (65.6–70.7) [3,193]	0.55 (0.43–0.70)	0.56 (0.43–0.74)
Women without a history of cancer	66.4 (65.5–67.3) [25,258]	0.51 (0.41–0.63)	0.51 (0.40–0.66)
**Ever had clinical breast exam**			
Breast cancer survivors	98.1 (97.1–99.1) [1,277]	1 [Reference]	
Female survivors of other cancers	93.2 (91.9–94.5) [3,314]	0.26 (0.15–0.47)	0.23 (0.11–0.49)
Women with no history of cancer	93.4 (93.0–93.9) [27,402]	0.28 (0.16–0.47)	0.20 (0.10–0.40)
**Last clinical breast exam < 1 year**			
Breast cancer survivors	84.2 (81.0–87.4) [1,228]	1 [Reference]	
Female survivors of other cancers	70.5 (67.9–73.0) [3,016]	0.45 (0.34–0.59)	0.45 (0.33–0.61)
Women with no history of cancer	68.6 (67.8–69.5) [24,910]	0.41 (0.32–.053)	0.36 (0.27–0.49)
**Cervical cancer screening^i^**			
**Pap test < 3 years**			
Breast cancer survivors	89.4 (85.9–93.0) [517]	1 [Reference]	
Female survivors of other cancers	84.2 (81.8–86.7) [1,771]	0.63 (0.42–0.96)	0.56 (0.35–0.90)
Women with no history of cancer	85.1 (84.4–85.8) [23,827]	0.67 (0.46–0.98)	0.47 (0.31–0.73)
**Colorectal cancer screening^j^**			
**Ever had blood stool test **			
Breast cancer survivors	44.9 (40.6–49.2) [1,210]	1 [Reference]	
Female survivors of other cancers	45.5 (42.7–48.3) [2,952]	0.78 (0.57–1.07)	1.12 (0.88–1.41)
Women with no history of cancer	38.2 (37.2–39.1) [21,482]	0.37 (0.28–0.49)	0.90 (0.73–1.10)
**Last blood stool test** <**1 year**			
Breast cancer survivors	10.7 (8.2–13.2) [1,277]	1 [Reference]	
Female survivors of other cancers	8.5 (7.1–9.9) [3,502]	0.78 (0.57–1.07)	1.08 (0.75–1.56)
Women with no history of cancer	4.3 (4.0–4.5) [34,044]	0.37 (0.28–0.49)	0.98 (0.72–1.34)
**Ever had sigmoidoscopy/colonoscopy**			
Breast cancer survivors	79.9 (76.6–83.3) [1,213]	1 [Reference]	
Female survivors of other cancers	76.7 (74.2–79.1) [2,971]	0.83 (0.64–1.06)	0.84 (0.63–1.11)
Women with no history of cancer	65.1 (64.2–66.1) [21,520]	0.47 (0.38–0.58)	0.48 (0.38–0.62)
**Most recent sigmoidoscopy^k^**			
Breast cancer survivors	3.5 (2.2–4.8) [1,188]	1 [Reference]	
Female survivors of other cancers	4.5 (3.1–5.9) [2,911]	1.29 (0.79–2.13)	1.14 (0.69–1.88)
Women with no history of cancer	3.7 (3.3–4.1) [21,187]	1.06 (0.72–1.56)	1.17 (0.76–1.79)
**Last sigmoidoscopy exam <5 years^k^**			
Breast cancer survivors	1.9 (1.0–2.8) [1,164]	1 [Reference]	
Female survivors of other cancers	2.3 (1.6–3.1) [2,852]	1.21 (0.68–2.17)	1.14 (0.60–2.16)
Women with no history of cancer	2.1 (1.8–2.4) [20,813]	1.08 (0.66–1.78)	1.06 (0.61–1.82)
**Most recent colonoscopy^k^**			
Breast cancer survivors	76.1 (72.6–79.7) [1,188]	1 [Reference]	
Female survivors of other cancers	71.7 (69.1–74.4) [2,911]	0.80 (0.63–1.01)	0.85 (0.65–1.10)
Women with no history of cancer	61.0 (60.0–62.0) [21,187]	0.49 (0.40–0.60)	0.51 (0.41–0.63)
**Last colonoscopy exam <10 years ago^k^**			
Breast cancer survivors	75.4 (71.7–79.0) [1,154]	1 [Reference]	
Female survivors of other cancers	70.8 (68.0–73.5) [2,821]	0.80 (0.64–0.99)	0.85 (0.65–1.11)
Women with no history of cancer	60.0 (59.0–61.0) [20,643]	0.53 (0.44–0.64)	0.51 (0.41–0.64)

Abbreviation: CI, confidence interval.

a Women with 1 cancer only, excludes women with multiple types of cancer.

b Excludes breast cancer survivors.

c The 9 states are Alaska, Connecticut, Indiana, Massachusetts, Missouri, New Mexico, Ohio, South Dakota, and Wisconsin, and the US territory is the Virgin Islands.

d Missing and unknown excluded.

e Adjusted for age, race, education and household income.

f Has smoked at least 100 cigarettes in lifetime and now smokes every day or some days.

g Consuming more than 1 drink per day.

h Women aged ≥40.

i Women aged 21 to 65.

j Women aged ≥50.

k Among those who ever had a sigmoidoscopy or colonoscopy.

Among women aged 40 or older, more than 90% in each group reported having mammography during their lifetime; however, breast cancer survivors had a significantly higher prevalence of ever having mammography (99.8%; 95% CI, 99.5%–100.0%) than other cancer survivors (95.7%; 95% CI, 94.5%–96.9%) and women without a history of cancer (91.3%; 95% CI,: 90.8%–91.9%) (Table 3). The prevalence of mammography in the prior year was significantly higher for breast cancer survivors (79.5%; 95% CI, 76.0%–83.0%) than for other cancer survivors (68.1%; 95% CI, 65.6%–70.7%) and women with no history of cancer (66.4%; 95% CI, 65.5%–67.3%). In addition, breast cancer survivors had a significantly higher prevalence of having a clinical breast examination within the previous year (84.2%; 95% CI, 81.0%–87.4%) than other cancer survivors (70.5%; 95% CI, 67.9%–73.0%) and women with no history of cancer (68.6%; 95% CI, 67.8%–69.5%) (Table 3). Furthermore, among women aged 21 to 65, breast cancer survivors were significantly more likely to have had a Pap test within the preceding 3 years (89.4%; 95% CI, 85.9%–93.0%) than women with no history of cancer (85.1%; 95% CI, 84.4%–85.8%).

Breast cancer survivors had a significantly higher prevalence of ever having a lower endoscopy (ie, sigmoidoscopy or colonoscopy; 79.9%; 95% CI, 76.6%–83.3%) than the women with no history of cancer (65.1%; 95% CI, 64.2%–66.1%) and a higher prevalence of having had a colonoscopy in the past 10 years (75.4%; 95% CI, 71.7%–79.0%) than women with no history of cancer (60.0%; 95% CI, 59.0%–61.0%).

In a subanalysis, the prevalence of never having a colonoscopy or not having a colonoscopy in the previous 10 years was stratified by sociodemographic characteristics. Breast cancer survivors with a household income of less than $25,000 or without health insurance were significantly less likely to be in compliance with colonoscopy screening guidelines (within the previous 10 years).

## Discussion

This study found that breast cancer survivors were significantly more likely to have had mammography, Pap tests, and colonoscopy, and were less likely to be current smokers. The results of this study align with the findings from previous breast cancer survivorship studies comparing female breast cancer patients and women with no history of cancer (18–20), and they expand the knowledge base with new information regarding risk factors, preventive practices, and survivorship health care among women with other types of cancer.

The American Society of Clinical Oncology (ASCO) recommends that female breast cancer survivors treated with breast-conserving therapy have their first post-treatment mammogram 1 year after the initial mammogram and no earlier than 6 months after completion of radiation therapy. Thereafter, unless otherwise indicated, a yearly mammographic evaluation should be performed (21). In this study, among women aged 40 or older, breast cancer survivors had a significantly higher prevalence of adherence to guidelines of mammography screening in the previous year than women in the other 2 groups. However, more than 20% of breast cancer survivors did not have a mammogram in the previous year.

Regular gynecologic follow-up is recommended for all women according to the ASCO breast cancer follow-up guidelines (21), and the Healthy People 2020 goal is for 93% of women aged 21 to 65 to have had a Pap test within the past 3 years (22). In this study, breast cancer survivors were significantly more likely to have had a Pap test within the preceding 3 years (89.4%) than other cancer survivors (84.2%) and women with no history of cancer (85.1%). However, there is still room for improvement among all 3 groups to achieve the Healthy People 2020 goal.

In this study, breast cancer survivors also had the highest prevalence of colonoscopy, and after we adjusted for sociodemographic factors, their rate remained significantly higher than that of women without a history of cancer. In this study, 75.4% of female breast cancer survivors had a colonoscopy in the previous 10 years, a rate that exceeds the Healthy People 2020 target of 70.5% for colorectal cancer screening (22) and approaches the goal of 80% by 2018 established by the National Colorectal Cancer Roundtable (23).

The finding that female breast cancer survivors had a significantly lower prevalence of smoking (10.3%) than other female cancer survivors (20.8%) is consistent with the findings of Mayer et al (20). Studies concur that cigarette smoking is associated with an increased risk of squamous cell cancer of the cervix (24,25). Mayer et al (20) also found a high prevalence of current smoking (48.9%) among women with cervical cancer. The high current smoking prevalence among women with other types of cancer in this study may be attributable to the large proportion of women with a history of cervical cancer in this group (15.4%).

Although research indicates that physical activity after a diagnosis of breast cancer may be beneficial in improving quality of life and reducing fatigue (26), a significantly higher proportion of female breast cancer survivors did not engage in leisure-time physical activity than the proportion of women with no history of cancer but the difference was not significant from the proportion for women with other types of cancer. These findings seem to concur with the finding that breast cancer survivors are more sedentary than noncancer control women (27) and may benefit from interventions promoting greater physical activity. Twice as many breast cancer survivors (15.1%) as female survivors of other cancer types (7.5%) reported current physical pain, which may contribute to the limited physical activity by both groups. The lower percentage of pain among survivors of other cancer types can be partly explained by the high proportion of skin cancer survivors (excluding melanoma, 28.2%) in this group because non-melanoma skin cancer seldom spreads to other parts of the body and treatment tends to be limited to the involved local site and not systemic treatment as in most other cancer types.

Consistent with other studies, our study found that most breast cancer survivors had health insurance (18,19) and that most of their health care was provided by primary care providers (28,29). This study showed that only about one-third of cancer survivors not currently in treatment (39.3% of women with breast cancer and 27.8% of women with other types of cancer) received a written cancer treatment summary and about two-thirds (62.3% of breast cancer survivors and 63.7% of women with other types of cancer) received written instructions on routine cancer check-ups, which is consistent with the findings of a previous study (30). In addition, only a small proportion of each cancer survivor group had participated in a clinical trial (8.1% of breast cancer survivors and 4.2% of other cancer survivors**)**.

The findings of this study have limitations. First, the BRFSS is subject to self-report measurement bias, selection bias if a portion of the population did not have landline telephone coverage, and nonresponse bias if nonresponders differ significantly from responders. Cellular telephones were also not included in the 2010 BRFSS but have since been incorporated. Second, cervical cancer may be overreported because of the similarities in treating cervical cancer precursors and cervical intraepithelial neoplasia. Third, diagnostic tests rather than screening tests may have been reported by some respondents, possibly leading to overestimates of screening. Fourth, the consumption of fruits and vegetables and types and levels of physical activity were assessed through the BRFSS every odd year, so these data were not available. Finally, cancer survivors who may have advanced disease and be too ill to participate in the survey or be residing in nursing homes, long-term care facilities, or hospice are not included and therefore may limit the generalizability of these results. Nevertheless, the BRFSS provides the largest sample for estimates of risk factors available because most cancer registries do not contain these data.

This study found that although a large proportion of breast cancer survivors receive regular mammography, Pap tests, and colonoscopy screening, there are significant opportunities for improvement to meet the national health objectives. The study also found that some breast cancer survivors continue to smoke, even though the prevalence is lower than for women with other types of cancer and women with no history of cancer. Therefore, the smoking status of each patient must be assessed, and effective smoking cessation interventions must be made available. These findings also underscore the need for pain management for a portion of survivors and the need for more research to identify effective methods to promote greater physical activity. Written treatment summaries and follow-up instructions establish communication between the cancer specialist, the primary care provider, and the patient to promote and enhance survivorship care coordination and self-management. These tailored-to-risk care plans should be developed for each survivor to ensure that all of the survivors’ needs are met. Findings of this study can be used by public health and health care providers to improve cancer survivorship care and management.
